# Intensification to biphasic insulin aspart 30/70 (BIAsp 30, NovoMix® 30) can improve glycaemic control in patients treated with basal insulins: a subgroup analysis of the IMPROVE™ observational study

**DOI:** 10.1111/j.1742-1241.2009.02064.x

**Published:** 2009-06

**Authors:** J Gumprecht, M Benroubi, V Borzi, R Kawamori, J Shaban, S Shah, M Shestakova, Y Wenying, R Ligthelm, P Valensi

**Affiliations:** 1 Department of Internal Diseases, Diabetology and Nephrology, Medical University of SilesiaZabrze, Poland; 2 Diabetes Centre, General Hospital of Athens “POLYKLINICI”Athens, Greece; 3 Department of Internal Medicine, Vittorio Emanuele HospitalCatania, Italy; 4 Department of Metabolism and Endocrinology, Juntendo University School of MedicineTokyo, Japan; 5 Endocrinology and Metabolism, Windsor Regional HospitalWindsor, ON, Canada; 6 Department of Endocrinology, Bhatia HospitalMumbai, India; 7 Institute of Diabetes, Federal Scientific Centre of EndocrinologyMoscow, Russia; 8 Department of Endocrinology, China-Japan Friendship HospitalBeijing, China; 9 EHM Clinic, HoofddorpRotterdam, The Netherlands; 10 Department of Endocrinology-Diabetology-Nutrition, Jean Verdier Hospital, AP-HP, Paris Nord UniversityCRNH-IdF, Bondy, France

## Abstract

**Aims:**

The international IMPROVE™ observational study investigated the safety profile and effectiveness of biphasic insulin aspart 30/70 (BIAsp 30) in the routine treatment of patients with type 2 diabetes. We present analyses for the subgroup of patients who switched from basal insulin to BIAsp 30.

**Methods:**

Patients in routine care who started insulin therapy with or switched to BIAsp 30 from existing insulin regimens were eligible for this 26-week study. This analysis includes only patients previously treated with basal insulin. Outcomes including adverse events, hypoglycaemic events and glycaemic profile were recorded from patients’ notes, recall and diaries.

**Results:**

Of the 748 patients included (age 59.7 ± 11.8 years, diabetes duration 11.4 ± 7.3 years, baseline HbA_1c_ 9.1 ± 1.6%), 497 were previously using human neutral protamine Hagedorn (NPH) insulin and 245 analogue basal insulin. Overall, major and minor hypoglycaemia rates decreased from baseline to final visit (major: 0.171 to 0.011; minor: 9.70 to 5.89 events/patient-year) and were similar between the subgroups. HbA_1c_ and fasting blood glucose were significantly reduced from baseline (NPH prestudy: −1.6%, −2.4 mmol/l; analogue basal prestudy: −1.8%, −2.4 mmol/l), as was postprandial blood glucose, with 33.8% of patients achieving the HbA_1c_ target < 7% without hypoglycaemia. Insulin dose increased slightly from prestudy (0.33 ± 0.21 U/kg), baseline (0.40 ± 0.20 U/kg) to final visit (0.52 ± 0.26 U/kg); most patients (76%) followed a twice-daily regimen at final visit. Body weight did not change significantly and treatment satisfaction increased.

**Conclusions:**

Patients with type 2 diabetes inadequately controlled on basal insulins may improve their glycaemic control by intensification to BIAsp 30 therapy.

**What’s known**

Intensification from basal insulin regimen to biphasic insulin aspart 30/70 (BIAsp 30) can result in improved glycaemic control in patients with type 2 diabetes as BIAsp 30 covers both basal and prandial insulin needs.

**What’s new**

- Here, we present results from a subgroup of inadequately controlled patients previously treated with basal insulins who intensified their therapy to BIAsp 30 and improved their glycaemic control, in many cases without hypoglycaemia.
- Patients were more satisfied with BIAsp 30 treatment than with their previous regimen.

## Introduction

The first line of treatment for type 2 diabetes usually involves lifestyle changes including diet and exercise (1) as well as oral antidiabetic drugs (OADs). However, as a result of the progressive nature of the disease, many patients require insulin therapy to control their blood glucose levels effectively (2,3) and minimise the risk of long-term complications. It is therefore important to initiate insulin therapy early in the disease process (4–6). The simplest and often popular way of initiating insulin is to start with basal insulin therapy (7). Basal insulin therapy, with or without OADs, can be an effective treatment option with just one daily injection and this simple regimen allows patients to adjust to a major change in the management of their diabetes.

Modern basal insulin analogues are effective in reducing HbA_1c_ and have also shown an improved safety profile compared with human insulins (8). However, insulin titration, or even changing the type of insulin, may be critical for the achievement of adequate glycaemic control (9). While basal insulin may be a good option for starting insulin treatment in some patients, patients’ needs change over time and glycaemic control (especially after meals) may become inadequate with basal only therapy (10); at this point the therapy should be intensified to either a basal–bolus or a premixed insulin regimen. Premixed insulin analogues offer the advantage of fewer daily injections than basal–bolus regimens as they provide both intermediate and rapid-acting components for basal and prandial insulin needs (11).

Biphasic insulin aspart 30/70 (BIAsp 30) is a premixed insulin analogue containing 30% soluble, rapid-acting insulin aspart and 70% intermediate-acting protamine-bound aspart in each injection. Several clinical trials have shown that initiating with or switching to BIAsp 30 therapy can achieve better glycaemic control than basal insulin therapy (9,12–15).

In addition, data from observational studies in diabetes provide valuable information as they complement results from randomised controlled trials (RCTs) and can indicate whether the benefits associated with particular treatments in RCTs translate into ‘real-life’ clinical practice (16,17). Results of one such observational study (PRESENT) have suggested that when patients are failing to reach glycaemic targets using basal insulin, they can improve their glycaemic control by intensifying their therapy to BIAsp 30 (18).

The IMPROVE™ study is a multinational observational study – the largest dataset to date – investigating the safety profile and effectiveness of BIAsp 30 in the treatment of type 2 diabetes (19). Here, we analysed safety and effectiveness results of BIAsp 30 treatment in patients who used basal insulin regimens before enrolling in the study and intensifying to BIAsp 30.

## Methods

### Study design

IMPROVE™ is a 26-week, open-label, non-randomised, multicentre observational study of patients with type 2 diabetes conducted in 11 countries (Canada, China, Greece, Gulf region, India, Iran, Italy, Japan, Poland, Russia and South Korea). Any patient with type 2 diabetes prescribed BIAsp 30 in routine clinical practice was eligible for the study. The details of the of IMPROVE™ study design have been published elsewhere (19).

In this paper, we report results of a subgroup of patients previously treated with basal insulins (human or analogue) with or without OADs. BIAsp 30 was prescribed as part of routine care once (qd), twice (bid) or three times daily (tid) depending on the patient’s needs. The dose and timing of BIAsp 30 treatment and of any concomitant medication were at the discretion of the physician. The dose was adjusted individually and any changes in BIAsp 30 treatment were recorded at the follow-up visit (at 3 months) and the final visit (at 26 weeks). The study was conducted in accordance with the Declaration of Helsinki. Procedures complied with local regulations governing observational studies, which were applicable to health authority and ethics committee approval and patient informed consent. Physicians received remuneration according to local regulations for the time spent collecting patient data.

### Outcome measures

The primary outcome measure was the incidence of major hypoglycaemic events reported as serious adverse drug reactions (SADRs). The secondary outcome measures included SADRs, number of major and minor hypoglycaemic events, changes in weight and body mass index (BMI), HbA_1c_, proportions of patients reaching a target of HbA_1c_ < 7.0%, fasting blood glucose (FBG), postprandial blood glucose (PPBG) after all main meals and treatment satisfaction as measured by the Diabetes Medication Satisfaction (DiabMedSat) questionnaire (20).

The full analysis set (FAS) included all patients with a baseline visit and at least one BIAsp 30 dose. The efficacy analysis set was defined as above but with at least one measurement of a hypoglycaemic event, blood glucose, weight or HbA_1c_ at baseline and final visit. Major hypoglycaemia was defined as an event with severe central nervous system symptoms that could not be self-treated, with either blood glucose levels < 2.8 mmol/l or symptoms that were reversed with either carbohydrate intake or glucagon or intravenous glucose administration. Minor hypoglycaemic events were defined as either symptoms of hypoglycaemia with blood glucose levels < 2.8 mmol/l that could be self-treated, or any asymptomatic blood glucose measurement < 2.8 mmol/l (19). Major hypoglycaemic events were recorded over 13 weeks prior to each visit and minor hypoglycaemic events over 4 weeks prior to each visit; both were then calculated as events per patient-year.

### Statistical analyses

Statistical comparisons of BIAsp 30 outcome measures at baseline and final visit were performed with paired *t*-tests for continuous variables and with Wilcoxon signed-rank tests for discrete variables. All testing used two-sided tests with the criteria set at α = 0.05.

## Results

### Patients

A summary of patient demographics is shown in Table 1. Of the total 748 patients included, 66.4% were using human neutral protamine Hagedorn (NPH) insulin ± OADs (*n*= 497), 21.0% insulin glargine ± OADs (*n*= 157) and 11.8% insulin detemir ± OADs (*n*= 88). Six patients (0.8%) were using other basal insulins or combinations (NPH plus glargine or detemir) and were excluded from the analysis, as they did not fit the predefined groups. The majority of patients (87%) were using one or more OADs before the baseline visit.

**Table 1:** Patient demographics

	Prestudy therapy		
Demographic	All patients using basal insulin (*n*= 748)	Human insulin (*n*= 497)*	Analogue insulin (*n*= 245)*
Age (years)	59.7 ± 11.8	60.5 ± 11.7	58.0 ± 11.9
Gender, M/F (%)	48/52	44/56	56/44
Weight (kg)	80.8 ± 19.6	81.2 ± 19.7	80.2 ± 19.3
BMI (kg/m^2^)	29.7 ± 6.9	30.0 ± 7.1	29.3 ± 6.4
Duration of diabetes (years)	11.4 ± 7.3	11.4 ± 7.0	11.4 ± 7.9
HbA_1c_ (%)	9.1 ± 1.6	9.0 ± 1.5	9.3 ± 1.8
Patients with/without OADs prestudy (%)	86.9/13.1	87.1/12.9	87.8/12.2

Data are mean (±SD) unless stated otherwise. *Six patients had other combinations of insulin and were excluded from the analyses. BMI, body mass index; OADs, oral antidiabetic drugs.

### Safety

**Figure 1:**
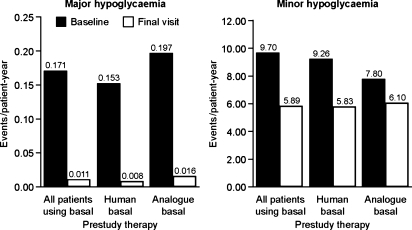
Rates of major and minor hypoglycaemia at baseline and final visit, according to prestudy basal insulin therapy

Of the 748 patients included in the safety analysis (FAS with final visit), only one patient (0.13%) reported a hypoglycaemic event as an SADR during the study. The proportion of patients reporting major hypoglycaemic events declined from 2.4% at baseline to 0.3% after 26 weeks and fewer patients reported minor hypoglycaemic events at the end of the study (17%) compared with baseline (27%). Hypoglycaemia rates and reductions were very similar for patients coming from human or analogue basal prestudy therapy (Figure 1).

### Effectiveness

All measures of glycaemic control – HbA_1c_, FBG and PPBG concentrations following breakfast, lunch and dinner – significantly improved after 26 weeks of BIAsp 30 treatment (p < 0.001) (Table 2). The mean HbA_1c_ reduction was 1.7%, FBG reduction was 2.35 mmol/l and PPBG reduction after breakfast was 4.36 mmol/l over the study period. Furthermore, 39.0% of patients achieved the HbA_1c_ targets of < 7%. The patients who achieved this target without hypoglycaemia (*n*= 253; 33.8%) had lower HbA_1c_ at baseline (8.58 ± 1.65%) and final visit (6.40 ± 0.40%) (change −2.17 ± 1.69%) compared with the total group. The changes in glycaemic measures were similar for patients switching from both human and analogue basal insulins (Table 2). What is more, patients in the qd prestudy basal insulin group had a similar mean HbA_1c_ reduction after switching to BIAsp 30 to those in the bid prestudy basal insulin group (−1.70%, −1.57% respectively).

**Table 2:** Change from baseline in effectiveness parameters when using BIAsp 30 for 6 months

	Prestudy therapy		
Outcome measure	All patients using basal insulin (*n*= 748)	Human insulin (*n*= 497)	Analogue insulin (*n*= 245)
**HbA**_**1c**_**(%)**			
Baseline	9.11 ± 1.63	8.97 ± 1.48	9.31 ± 1.80
Final visit	7.39 ± 1.16	7.34 ± 1.06	7.48 ± 1.32
Change from baseline	−1.72 ± 1.58***	−1.64 ± 1.44***	−1.83 ± 1.72***
**Patients reaching**			
**HbA_1c_ < 7.0% (%)**	39	40.7	34.8
**FBG (mmol/l)**			
Baseline	9.45 ± 2.51	9.54 ± 2.43	9.32 ± 2.65
Final visit	7.10 ± 1.99	7.18 ± 2.13	6.94 ± 1.69
Change from baseline	−2.35 ± 2.97***	−2.36 ± 3.00***	−2.38 ± 2.92***
**PPBG breakfast (mmol/l)**			
Baseline	12.88 ± 3.69	12.56 ± 3.54	13.59 ± 3.89
Final visit	8.51 ± 2.17	8.28 ± 2.15	8.98 ± 2.17
Change from baseline	−4.36 ± 3.30***	−4.28 ± 3.23***	−4.60 ± 3.42***
**PPBG lunch (mmol/l)**			
Baseline	11.94 ± 3.36	11.65 ± 3.15	12.57 ± 3.69
Final visit	8.35 ± 1.61	8.14 ± 1.38	8.78 ± 1.93
Change from baseline	−3.59 ± 3.30***	−3.52 ± 3.12***	−3.79 ± 3.64***
**PPBG dinner (mmol/l)**			
Baseline	11.64 ± 2.75	11.61 ± 2.72	11.68 ± 2.79
Final visit	8.20 ± 1.94	8.06 ± 1.64	8.63 ± 2.64
Change from baseline	−3.44 ± 2.82***	−3.55 ± 2.70***	−3.06 ± 3.11***
**BIAsp 30 daily dose (U/kg)**			
Baseline	0.40 ± 0.20	0.39 ± 0.20	0.40 ± 0.20
Final visit	0.53 ± 0.26	0.54 ± 0.25	0.50 ± 0.27
Change from baseline	0.13 ± 0.20***	0.14 ± 0.20***	0.10 ± 0.20***
**Weight (kg)**			
Baseline	80.77 ± 19.63	81.16 ± 19.69	80.21 ± 19.34
Final visit	80.74 ± 19.24	81.26 ± 19.38	79.91 ± 18.78
Change from baseline	−0.03 ± 4.38 ns	−0.10 ± 3.49 ns	−0.30 ± 5.82 ns

Values are mean (± SD). ***p < 0.0001; ns, not significant; FBG, fasting blood glucose; PPBG, postprandial blood glucose; BIAsp 30, biphasic insulin aspart 30/70.

### Weight

There was no significant weight change from baseline (80.77 ± 19.6 kg) to final visit (80.74 ± 19.2 kg; change −0.03 ± 4.4 kg) and BMI was also stable (29.69 kg/m^2^ at both baseline and final visit). Patients switching from both human and analogue basal insulins showed similar results (Table 2).

### BIAsp 30 dose and injection frequency

The mean total insulin daily dose increased from prestudy (0.33 ± 0.21 U/kg), baseline (0.40 ± 0.20 U/kg) to final visit (0.53 ± 0.26 U/kg). Patients using qd basal insulin prestudy (*n*= 437) started on a slightly lower dose than those using bid basal insulins (*n*= 289) (0.36 vs. 0.44 U/kg respectively). However, the BIAsp 30 dose increased similarly in both cases by the end of the study (0.50 vs. 0.57 U/kg; dose change 0.14 vs. 0.13 U/kg respectively). For patients who achieved an HbA_1c_ target of < 7% without hypoglycaemia, the mean total BIAsp 30 daily dose increased from 0.37 ± 0.18 U/kg at baseline to 0.46 ± 0.22 U/kg at final visit.

Prior to the study, 58.4% of patients were using a qd basal insulin regimen (*n*= 437); 38.6% (*n*= 289) a bid regimen; 1.7% (*n*= 13) used insulin tid and 1.2% (*n*= 9) injected insulin four times per day. At baseline, the majority of patients started using BIAsp 30 bid (*n*= 612, 81.8%); 12.0% (*n*= 90) used BIAsp 30 qd and 6.1% (*n*= 46) injected BIAsp tid. By the end of the study, 75.8% of patients (*n*= 567) were still using BIAsp 30 bid, a qd BIAsp 30 regimen was used by 6.3% of patients (*n* = 47) and a tid regimen by 17.8% of patients (*n*= 133). At the final visit, 62% of patients were still using one or more OADs.

### Patient satisfaction

At baseline, only 12.2% of patients were very or extremely satisfied with their current diabetes treatment; at final visit this proportion increased to 59.7%. The corresponding proportions for those switching from human and analogue basal insulins were 11.6 to 61.0% and 13.6 to 55.4% respectively.

## Discussion

The results of this subgroup analysis of the IMPROVE™ study suggest that by intensifying basal insulin regimens to a BIAsp 30 regimen in routine care, glycaemic control can be significantly improved in inadequately controlled patients with type 2 diabetes. Furthermore, the improved glycaemic control was achieved with a reduced risk of both major and minor hypoglycaemic events and with no significant change in weight. All these factors contributed to the increased patient treatment satisfaction following a switch from basal insulin to BIAsp 30. The DiabMedSat questionnaire, which was used to assess treatment satisfaction, is a tool which integrates measures for disease burden, symptom relief, treatment burden and medication satisfaction (20). The greater overall satisfaction with diabetes medication that we report for BIAsp 30 compared with the previous insulin therapy is thus a clear indication of improvements in many aspects of patients’ lives.

The improvement seen in this cohort with poor glycaemic control at baseline indicates that a more intensive insulin therapy is not only appropriate, but long overdue. These patients previously using basal insulin (almost 90% were also taking OADs) had been diagnosed, on average, over 11 years previously and had mean HbA_1c_ levels above 9%. Long-term hyperglycaemia will bring an increased risk of diabetic complications (2), so further intervention was certainly required in these patients. Basal insulins can be effective when glycaemic control is particularly poor, but their benefit reaches a ceiling when HbA_1c_ reaches about 8.5% because at this point postprandial hyperglycaemia is the main contributor to glycaemic load (10). Switching to BIAsp 30, comprising rapid-acting and basal components, was therefore an appropriate intensification insulin choice for these patients.

Results similar to those we show have been reported by the PRESENT observational study of BIAsp 30: patients coming from basal insulin analogue therapy achieved a mean HbA_1c_ reduction of 1.6% and those previously treated with human basal insulins a reduction of 1.4% (both p < 0.0001). Our figures were 1.8% and 1.6% respectively, slightly lower than the mean HbA_1c_ reduction for the global cohort (−2.3%, *n*= 52,419) (18). The improvements in FBG and PPBG in the IMPROVE™ study were also comparable with those of the PRESENT study. The change in FBG was −2.4 mmol/l for both basal insulin groups in the IMPROVE™ study and −2.8 and −3.7 mmol/l for the human and analogue basal insulin switchers respectively in PRESENT. As expected, PPBG decreased after breakfast and dinner; it also decreased after lunch despite no lunchtime injection in most patients. The lowered PPBG after breakfast may have also lowered prelunch blood glucose, thus leading to a lower absolute PPBG level at lunchtime, even if glucose excursions may have been similar at all time points.

It is very encouraging that almost 40% of patients in these analyses achieved target HbA_1c_ of < 7.0% and the majority of these did so without experiencing hypoglycaemia. This compares favourably with data from a RCT, in which 33% of patients with type 2 diabetes achieved this target using bid BIAsp 30 (approximately one-third of patients were previously treated with basal insulin) after 26 weeks of therapy (21). Interestingly, for patients who achieved the HbA_1c_ target of < 7% without hypoglycaemia, it seems that an absence of hypoglycaemia, coupled with a lower baseline HbA_1c_, enabled these patients to achieve a much lower final HbA_1c_ than the overall cohort (6.4% vs. 7.4% respectively), with similar mean doses of BIAsp 30.

Furthermore, the patients in the bid prestudy basal group did not experience a greater HbA_1c_ reduction after switching to BIAsp 30 than those in the qd prestudy basal group; therefore the absolute dose does not appear to be the key factor in achieving glycaemic targets. The current results suggest that dose titration allows patients to achieve targets, as the dose increment over the course of the study was very similar in both groups.

All patients who fail to achieve glycaemic control with basal insulin, with or without OADs, require additional treatment measures. Switching insulin therapy to BIAsp 30, which addresses both basal and prandial insulin needs, therefore constitutes treatment intensification (22). From the results we report here, we can draw some conclusions about how dose switching was implemented in real clinical practice. First, when patients were switched from basal insulin to BIAsp 30, most were started on a bid regimen; after 26 weeks, 12% of patients intensified therapy to BIAsp 30 tid. Secondly, patients who transferred from qd basal insulin to BIAsp 30 (mostly to bid) approximated a 1 : 1 basal insulin transfer and similarly those who transferred from bid basal insulin to BIAsp 30. These data may indicate a stepwise intensification from basal insulin, starting with a 1 : 1 BIAsp 30 qd dose switch, intensifying to BIAsp 30 bid for most patients. Thereafter, a small proportion were switched to BIAsp 30 tid within 6 months. The 1-2-3 study (9) of BIAsp 30 in patients with type 2 diabetes supports this progression. Here, 100 patients were started on BIAsp 30 qd and intensified to bid and tid at 16-week intervals if target HbA_1c_ (≤ 6.5%) was not achieved. In the 1-2-3 study, the proportions of patients that reached HbA_1c_ < 7.0% on qd, bid and tid BIAsp 30 were 41%, 70% and 77% respectively (9).

Observational studies offer the opportunity of studying large and heterogeneous populations; however, they also have some limitations. These include a lack of control groups, potential patient recall bias and possible variations in clinical practice between countries. The limitations of the IMPROVE™ study have been discussed at length in the article reporting baseline data (19).

## Conclusions

The results of this IMPROVE™ subgroup analysis demonstrate that patients with type 2 diabetes inadequately controlled on basal insulins may improve their glycaemic control by intensification to BIAsp 30 therapy. Regardless of their prior basal insulin regimen, switching to BIAsp 30 – bid in the majority of cases – enabled many patients in this international cohort to achieve the HbA_1c_ target without hypoglycaemia.
